# Inhibitory Receptors and Checkpoints in Human NK Cells, Implications for the Immunotherapy of Cancer

**DOI:** 10.3389/fimmu.2020.02156

**Published:** 2020-09-03

**Authors:** Simona Sivori, Mariella Della Chiesa, Simona Carlomagno, Linda Quatrini, Enrico Munari, Paola Vacca, Nicola Tumino, Francesca Romana Mariotti, Maria Cristina Mingari, Daniela Pende, Lorenzo Moretta

**Affiliations:** ^1^Department of Experimental Medicine (DIMES) and Center of Excellence for Biomedical Research, University of Genoa, Genoa, Italy; ^2^Department of Experimental Medicine, University of Genoa, Genoa, Italy; ^3^Department of Immunology, IRCCS Ospedale Pediatrico Bambino Gesù, Rome, Italy; ^4^Department of Pathology, IRCCS Sacro Cuore Don Calabria, Negrar, Italy; ^5^UOC Immunology, IRCCS Ospedale Policlinico San Martino, Genoa, Italy

**Keywords:** natural killer cells, inhibitory NK receptors, immune checkpoints, tumor immunotherapy, tumor escape

## Abstract

The highly destructive mechanisms by which the immune system faces microbial infections is under the control of a series of inhibitory receptors. While most of these receptors prevent unwanted/excessive responses of individual effector cells, others play a more general role in immunity, acting as true inhibitory checkpoints controlling both innate and adaptive immunity. Regarding human NK cells, their function is finely regulated by HLA-class I-specific inhibitory receptors which allow discrimination between HLA-I^+^, healthy cells and tumor or virus-infected cells displaying loss or substantial alterations of HLA-I molecules, including allelic losses that are sensed by KIRs. A number of non-HLA-specific receptors have been identified which recognize cell surface or extracellular matrix ligands and may contribute to the physiologic control of immune responses and tolerance. Among these receptors, Siglec 7 (p75/AIRM-1), LAIR-1 and IRp60, recognize ligands including sialic acids, extracellular matrix/collagen or aminophospholipids, respectively. These ligands may be expressed at the surface of tumor cells, thus inhibiting NK cell function. Expression of the PD-1 checkpoint by NK cells requires particular cytokines (IL-15, IL-12, IL-18) together with cortisol, a combination that may occur in the microenvironment of different tumors. Blocking of single or combinations of inhibitory receptors unleashes NK cells and restore their anti-tumor activity, with obvious implications for tumor immunotherapy.

## Introduction

To combat infections, the immune system exploits highly destructive mechanisms. These mechanisms are triggered by an array of receptors that evolved during phylogenesis from structures ensuring phagocytosis and killing of invading pathogens toward highly sophisticated, clonally distributed, receptors encoded by rearranging genes. Remarkably, most of the “primitive” receptors did not disappear during evolution but rather co-evolved with adaptive immunity and are playing, in contemporary vertebrates, a synergistic role, contributing to improved anti-microbial responses. A good example is provided by the Fc-gamma receptors, evolved from a primitive surface protein into receptors recognizing IgG antibodies (Abs), allowing greater killing or phagocytosis of Ab-coated bacteria or target cells (1). In particular, NK cells, expressing the FcγRIIIa (also known as CD16), are considered the most important effectors of antibody-dependent cell-mediated cytotoxicity (ADCC) in humans.

The exploitation of highly destructive mechanisms to control infections would require means to avoid damages to healthy cells and, in general, to the whole organism. Thus, efficient mechanisms have been acquired to prevent damages to self by downmodulating immune responses and inflammation at the termination of infection. A major role in ensuring this crucial activity is played by an array of inhibitory receptors which may control the function of individual cells of both innate and adaptive immunity and, in some instances, may function as true checkpoints, ensuring a wide control of immune response and inflammation.

Focusing on human NK cells, they express different HLA class I–specific receptors that allow discrimination between healthy and virus-infected or tumor cells (2), while other receptors such as TIGIT and CD96, although controlling NK cell function, play a role also in the regulation of cell adhesion and migration/homing (3). In this context, studies of tissue distribution of their ligands, such as PVR (CD155) and Nectin-2 (CD112), may provide useful information on the possible migration/homing of cells expressing the corresponding receptor. Other receptors, such as CD69 and CD103, represent tissue retention receptors and may provide important markers to identify NK and T cells capable of infiltrating and staying in normal peripheral tissues or tumors (4).

In this contribution, we will delineate some of the main inhibitory receptors expressed by human NK cells. Remarkably, Killer Ig-like Receptors (KIR), discovered by Moretta et al. in 1990 (5, 6) are the prototype of the inhibitory receptors controlling cells of the immune system. These and other HLA class I-specific receptors provided the molecular basis of the “missing-self hypothesis” and explained how NK cells may discriminate between healthy and tumor or virus-infected cells (7). Moreover, NK cells can express several non-HLA-specific inhibitory receptors that contribute in regulating immune responses. Some inhibitory receptors are constitutively expressed by NK cells (such as KIR and NKG2A) and are involved in the regulation of NK cell tolerance against healthy tissues, while others (such as PD-1) are expressed at very low level in NK cells from healthy donors, but increase in pathological conditions. All these inhibitory receptors act as immune checkpoints regulating anti-tumor NK cell function by the recognition of specific ligands on tumor cells thus favoring tumor escape from NK cell cytotoxicity.

## HLA-Specific Inhibitory NK Receptors

**FIGURE 1:**
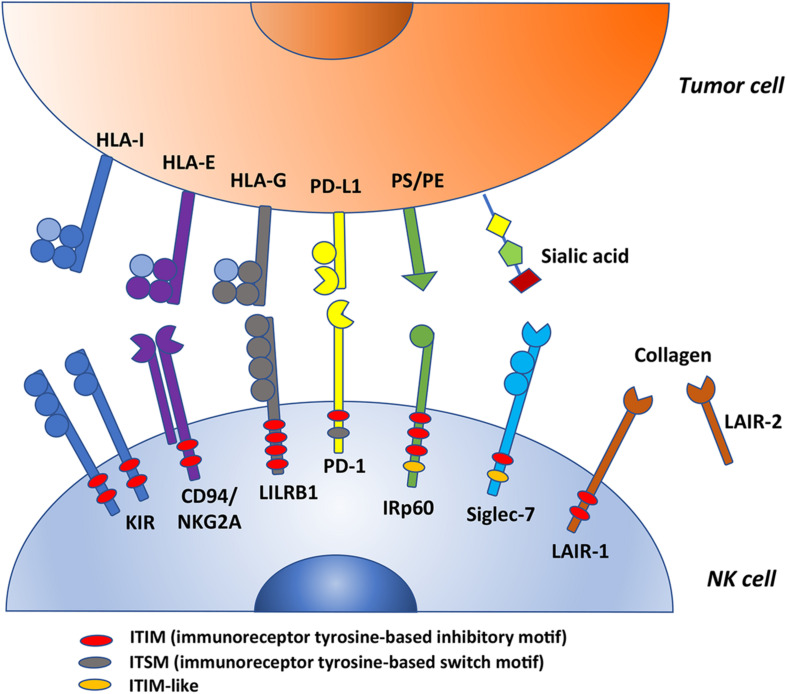
This figure summarizes some ITIM-bearing molecules expressed by human NK cells that could act as checkpoints in cancer immunotherapy. KIRs, CD94/NKG2A and LILRB1 are HLA-specific inhibitory receptors whereas PD-1, IRp60, Siglec-7 and LAIR-1 are non-HLA-specific inhibitory receptors (their ligands are indicated in the figure). All these molecules possess variable numbers and different types of ITIMs. In particular, PD-1 express one ITIM; KIRs, CD94/NKG2A and LAIR-1 have two ITIMs (among KIRs, only KIR3DL3 and KIR2DL4 express one ITIM); IRp60 has three ITIMs; LILRB1 has four ITIMs (indicated in red in the figure). In addition, PD-1 carries also an ITSM motif (gray) whereas IRp60 and LAIR-1 an ITIM-like motif (orange). PS, phosphatidylserine; PE, phoshatidylethanolamine.

In humans, the molecular basis for NK cell tolerance toward healthy autologous cells is provided by HLA-specific inhibitory receptors (iNKR), that are mainly represented by KIR, CD94:NKG2A, and LILRB1 (2, 6, 8–10). Inhibitory KIRs (iKIR), characterized by 2 or 3 Ig-like extracellular domains and a long cytoplasmic tail (KIR2DL, KIR3DL), recognize allotypic determinants shared by distinct groups of HLA class I molecules (KIR-ligands, KIR-L), as recently reviewed (11). CD94:NKG2A heterodimer, composed by C-type lectin-like proteins, is specific for the non-classical HLA-E molecules, that are stabilized by peptides mainly derived from the leader sequences of HLA-A, -B, or –C (12, 13). LILRB1 displays a broad specificity for HLA (14, 15). Upon receptor engagement, the immunoreceptor tyrosine-based inhibitory motifs (ITIM) become phosphorylated and recruit tyrosine phosphatases, thus delivering an inhibitory signaling cascade (16–18). Table 1 and Figure 1 summarize these receptor/ligand interactions.

**TABLE 1:** HLA-I specific and non-HLA-I specific inhibitory receptors, their distribution and ligands.

	Molecule	CD	Cell distribution	Ligand
HLA specific inhibitory receptors	KIR2DL1	CD158a	NK cells, T cells	HLA-C^K80^ allotypes (HLA-C2 epitope)
	KIR2DL2/3	CD158b1/b2	NK cells, T cells	HLA-C^N80^ allotypes (HLA-C1 epitope) HLA-B*46:01 and -B*73:01
	KIR2DL5	CD158f	NK cells, T cells	?
	KIR3DL1	CD158e1	NK cells, T cells	HLA-A Bw4, HLA-B Bw4
	KIR3DL2	CD158k	NK cells, T cells	HLA-A*03 and -A*11, HLA-F
	KIR3DL3	CD158z	NK cells, T cells	?
	LILRB1/LIR-1/ILT2	CD85j	NK cells, T cells, B cells, monocytes, DCs	HLA-G, various HLA-I allotypes
	NKG2A	CD159a	NK and T cells	HLA-E
	LAG-3	CD223	Activated NK cells, activated T cells, B cells, pDCs	HLA-II

Non-HLA specific inhibitory receptors	PD-1	CD279	NK cells, T cells, B cells, myeloid cells	PD-L1, PD-L2
	TIM-3	CD366	NK cells, T cells, DCs, monocytes, macrophages, mast cells	Gal-9, PS, HMGB1, CEACAM1
	TIGIT	NA	NK cells, T cells	CD155, CD112, CD113
	Tactile	CD96	NK cells, T cells	CD155, CD111
	Siglec-7/p75/AIRM-1	CD328	NK cells, T cells, granulocytes, monocytes,	Sialic acid
	Siglec-9	CD329	NK cells, T cells, B cells, granulocytes, monocytes	Sialic acid
	KLRG1	NA	NK cells, T cells	cadherins
	IRp60	CD300a	NK cells, T cells, B cells, neutrophils, eosinophils, mast cells, pDC	phosphatidylserine (PS), phoshatidylethanolamine (PE)
	LAIR-1/p40	CD305	NK cells, T cells, B cells, monocytes, granulocytes, DCs, mast cells, macrophages, CD34^+^ hematopoietic progenitor cells, thymocytes	Collagen, C1q, surfactant protein D
	CEACAM-1	CD66a	epithelial cells, various leukocytes	CEACAM-1, CEACAM-5
	NKRP1A	CD161	NK cells, T cells	LLT1
	IAP	CD47	NK cells, T cells, B cells, monocytes, macrophages, DCs, neutrophils	SIRP1a, TSP-1

During NK cell development, immature stages primarily express CD94:NKG2A, while KIRs are acquired upon maturation. NK cells go through a process termed “education,” involving the iNKR/self-HLA interaction, whose strength positively influences the functional potential of NK cells (19). Extremely diversified self-tolerant iNKR phenotypic repertoires can be observed on peripheral blood NK cell pool among the various individuals (17). This heterogeneity is primarily determined by the high polymorphism of the independently co-inherited *KIR* and *HLA* class I genotypes, and by the stochastic KIR expression pattern on NK cells (20). NK cells can be efficient even when expressing single-iKIR, provided that it strongly interacts with self-HLA. This NK cell can kill the pathological cell that has lost even a single-HLA allotype through the mechanism of “missing-self recognition.” Regarding CD94:NKG2A/HLA-E interaction, a dimorphism in *HLA-B* leader sequence at residue − 21 encoding either a good binding methionine (− 21 M) or a low binding threonine (− 21 T) determines the variability in HLA-E expression; NKG2A^+^ cells from individuals carrying at least one − 21 M *HLA-B* alleles are more educated (21). Consistent with this finding, in acute myeloid leukemia (AML) patients treated with immunotherapy, a better leukemia-free survival (LFS) was observed in patients with − 21 M/x than − 21 T/T *HLA-B* alleles (22).

In addition to genetics, environmental factors can impact on the receptor repertoire. The most remarkable example is represented by cytomegalovirus (CMV) infection, that promotes the expansion of functionally and phenotypically skewed NK cells with adaptive features through epigenetic alterations (23, 24). These cells are characterized by the expression of the activating CD94:NKG2C, mainly co-expressing KIR2DL specific for self-HLA-C allotypes, CD57 (a marker of terminally differentiation stage), and by the lack of NKG2A (25–27). Notably, in view of their long term persistence (28–30), expansion capabilities (31) and high ADCC abilities (32, 33), CMV-driven adaptive NK cells also represent a suitable target for anti-leukemia immunotherapeutic strategies (e.g., CD16-based immune engagers, adoptive cell transfer, CAR-engineering) (34).

KIRs have been shown to be clinically relevant in allogeneic hematopoietic stem cell transplantation (HSCT) to cure acute leukemia, in particular from HLA-haploidentical donors whose repertoire presents educated iKIR(s) that do not recognize the cognate KIR-L(s) in the recipient. When KIR/KIR-L mismatches in graft-versus-host (GvH) direction occur, alloreactive NK cells can be generated in the transplanted patient, with efficient anti-leukemia activity (35). This has been proven especially beneficial in acute myeloid leukemia (AML) adult patients (36), and in acute lymphoblastic leukemia (ALL) pediatric patients (37). Algorithms for donor selection criteria have been created, considering NK alloreactivity and KIR gene profiles, to improve the clinical outcome in HSCT (38–41).

A great improvement in cancer immunotherapy has been achieved with immune checkpoint inhibitors (ICI), by the use of therapeutic antibodies blocking inhibitory checkpoints. With the aim to potentiate/unleash the anti-tumor NK cell function, clinical grade monoclonal antibodies (mAbs) targeting KIR and NKG2A have been produced. Lirilumab (1-7F9, IPH2101), a first-in-class fully human IgG4 mAb targeting KIR2D, has been employed in phase I trials to treat hematological malignancies or solid tumors, also in association with Lenalidomide (as NK cell stimulant) in multiple myeloma, resulting to be safe but with low anti-tumor efficacy (42–44). More promising clinical results have been obtained with IPH4102 targeting KIR3DL2 on cutaneous T cell lymphoma, particularly in Sèzary syndrome (45). Of extreme interest for the clinical potential is monalizumab, a humanized IgG4-blocking anti-NKG2A mAb, that can unleash both NK and T-cell responses (46). Indeed, NKG2A/HLA-E interaction can downregulate anti-tumor immune responses. Clinical trials using monalizumab in combination with durvalumab (anti-PD-L1) for the treatment of solid tumors, and, especially, in combination with cetuximab (anti-EGFR) for the treatment of head and neck cancers, show clear signs of efficacy (46).

## Non-HLA-Specific Inhibitory NK Receptors

In addition to the HLA class I-specific receptors, NK cells express several other ITIM-containing receptors importantly contributing to regulate immune responses (Table 1) (47–60). We focus here on the critical immune checkpoint PD-1 and on Siglec-7/p75/AIRM1/CD328, LAIR-1/p40/CD305, and IRp60/CD300a, originally identified in our labs, representing additional immune checkpoints possibly dampening anti-tumor NK cell responses in given pathological settings (Figure 1). Siglec-7, IRp60 and LAIR-1 are rarely discussed in most reviews on immune checkpoints in NK cell context, however, they represent relevant receptors to target in anti-tumor immunotherapies. Indeed, their ligands are expressed or even upregulated on several tumors.

## PD-1

PD-1 is a type I transmembrane glycoprotein belonging to the CD28/CTLA4 subfamily of the Ig superfamily, containing an IgV-type extracellular domain (61). Its cytoplasmic domain contains an ITIM and an immunoreceptor tyrosine-based switch motif (ITSM) and, interestingly, the tyrosine residue in the ITSM, but not in the ITIM, is required for the inhibitory cascade (62). PD-1 expression was initially described on T, B, myeloid cells and, more recently, on NK cells (47). PD-1 ligands (PD-Ls, namely PD-L1 and PD-L2) are expressed by hematopoietic and non-hematopoietic cells and, importantly, they are often expressed by tumor cells. Indeed, while in normal conditions PD-1/PD-L axis regulates peripheral tolerance, in the context of cancer it represents a mechanism of “escape” from immune system and in particular from PD-1-expressing cytotoxic lymphocytes (63). In addition to PD-1-expressing CD8^+^ T cells, also PD-1^+^ NK cells have been identified in several tumors, including multiple myeloma, Kaposi sarcoma, ovarian carcinoma, digestive and lung cancer (31, 47, 64–66). Differently from T cells, which are induced to upregulate PD-1 expression upon activation, NK cells from the peripheral blood of healthy donors do not express PD-1 on their surface, with the exception of a minor fraction of CMV seropositive individuals (47). Human NK cells have been shown to display an intra-cytoplasmatic pool of PD-1 mRNA and protein localized in the Golgi (67). An analysis of pleural effusion contents from primary and metastatic tumors identified glucocorticoids as key components of the tumor microenvironment indispensable for PD-1 induction on NK cells surface, in combination with the signals from the cytokines IL-12, IL-15 and IL-18 (68). Glucocorticoids were shown to increase PD-1 expression at the transcriptional level in both human and murine NK cells (68, 69). In addition, in human CD56^*bright*^ NK cells, these hormones activate a transcriptional program responsible for enhanced translation and translocation of proteins to the plasma membrane, which indirectly contributes to increase PD-1 surface expression. Notably, PD-1^+^ NK cells are not exhausted, but show an impaired response specifically against PD-L1-expressing target cells (68).

Blockade of the PD-1/PD-L1 axis through monoclonal antibodies represents a major breakthrough in oncology, showing significant clinical success in the treatment of several types of cancers (70, 71). This blockade allows unleashing not only T cell-, but also NK cell-mediated anti-tumor response. This is relevant especially in the treatment of tumors that have lost HLA-I expression and are thus “invisible” to T cells. Despite its success, only one third of patients is responsive to anti-PD-1 immunotherapy (72). One important factor that may be responsible for this lack of response is represented by the misclassification of tumors in terms of PD-L1 expression. The immunohistochemical detection of this biomarker in tumor samples usually guides the decision of the appropriate therapeutic strategy, together with other parameters. PD-L1 expression heterogeneity, interclone differences among antibodies used for immunohistochemistry and inter/intra observer variability may explain why the rates of clinical response to treatment with PD-1/PD-L1 inhibitors do not always correlate with PD-L1 detected expression (73–76). Moreover, the recent identification of the molecular mechanisms driving PD-1 expression on NK cells suggests that including synthetic corticosteroids in the therapeutic regimen for cancer patients may be counterproductive in combination with the blockade of this checkpoint.

## SIGLEC-7/p75/AIRM1/CD328

Siglec-7 is a surface inhibitory receptor belonging to a family of Sialic acid recognizing Immunoglobulin-like Lectins (Siglecs) that is mostly confined to NK cells, but is expressed also on monocytes, a minor fraction of CD8^+^ T cells and granulocytes (48, 77). Siglec-7 was originally identified as a 75-kD glycoprotein, encoded by a gene located on chromosome 19 where most inhibitory receptors regulating NK-mediated cytotoxicity are found (i.e., KIRs, LILRB1, and LAIR-1) (48). In line with most inhibitory receptors, Siglec-7 is characterized by Ig-like domains in the extracellular portion and a classical ITIM, together with an ITIM-like domain, in its cytoplasmic tail, capable of switching off activating signals on NK cells (48). Siglec-7 preferentially binds to α2-6-linked sialic acids and to α2,8-disialic acid that is found on GD3 ganglioside (78).

Siglec-7, along with other Siglecs, can regulate immune responses contributing to immune tolerance, however, it can also decrease anti-tumor immunity on account of the aberrant expression of sialylated glycans on the surface of malignant cells of different histotypes [e.g., AML, CLL, melanoma, renal cell carcinoma, colon adenocarcinoma (79, 80)]. Indeed, hyper-sialylation represents a relevant tumor escape mechanism that can directly affect NK cell-mediated tumor killing, as demonstrated by reduced NK cell-cytotoxicity against tumors expressing Siglec-7 ligands. Remarkably, the employ of antibodies blocking Siglec-7 engagement could restore tumor lysis (80–82). Interestingly, Siglec-7 reduced expression represents a hallmark of CMV-driven adaptive NK cell subsets (32, 33) and could favor their cytotoxicity against HLA-I^*low/neg*^ tumors.

Based on the above observations, Siglec-7 represents an attractive immune checkpoint that can be targeted to enhance anti-tumor responses (83). In this context, besides anti-Siglec-7 blocking antibodies, different approaches have been proposed, including the employ of small soluble Siglec-7 ligands, designed to display high avidity for the receptor based on its crystal structure (84, 85). These molecules can increase NK-cell mediated tumor lysis although less efficiently than specific anti-Siglec-7 antibodies (86). Interestingly, a recent study showed that cells engineered with a Siglec-7-based CAR construct can display efficient anti-tumor activity both *in vitro* against several tumor cell lines expressing Siglec-7 ligands and *in vivo* in xenograft murine models (87).

## LAIR-1/p40/CD305

Another non-HLA-specific inhibitory NK receptor is represented by the Leukocyte-Associated Immunoglobulin-like Receptor-1 (LAIR-1) (88, 89), which is a type I transmembrane glycoprotein characterized by an extracellular C2-type Ig-like domain and two ITIMs in the cytoplasmic tail (90, 91).

LAIR-1 is one of the most widely distributed inhibitory receptors and could play a role in controlling various phases of the immune response. Indeed, it is expressed not only on NK cells but also on other cells of innate immunity (such as monocytes, granulocytes, dendritic cells, mast cells, macrophages) (90, 92–94), on T and B lymphocytes (49, 95, 96), on CD34^+^ hematopoietic progenitor cells (97) and on the majority of thymocytes (90). Interestingly, during the process of cell maturation and activation LAIR-1 expression is decreased on various immune cells (i.e., CD4^+^ T cells, neutrophils, B cells) (93, 96, 98).

The interaction between LAIR-1 and its several ligands, such as extracellular matrix collagens (99), the C1q complement component (100), and the surfactant protein D (101), induces phosphorylation of both ITIMs and inhibition of the immune cell activation or differentiation. In particular, the LAIR-1 cross-linking with monoclonal antibodies, or with its ligands, inhibits the NK and CTL cytotoxicity (102). LAIR-1-mediated inhibition occurs through SHP-1 and SHP-2, but also through the recruitment of Csk (103) that inactivates Src family kinases.

Remarkably, the upregulation of collagen expression by tumor cells and/or tumor stroma could lead to the downregulation of anti-tumor responses mediated by the inhibitory collagen receptor LAIR-1 expressed on NK cells and other effector immune cells.

LAIR-1 can be also detected in the supernatant of stimulated human lymphocytes, suggesting its shedding upon cellular activation (104). In the soluble form, LAIR-1 could interfere with the interaction between the transmembrane receptor and its ligands, thus restoring functions of immune cells.

A similar result could also occur with the LAIR-2 protein that is 84% homologous to LAIR-1 but lacks the transmembrane and intracellular domain. Indeed, the binding of LAIR-2 to collagens could efficiently block LAIR-1–collagen interaction (105). On this basis, an interesting approach has been developed to block immune suppression mediated by LAIR-1. It is based on the use of NC410, a novel reagent capable of mimicking the natural decoy effects of LAIR-2. The blockade of the LAIR-1-mediated inhibition by NC410 can restore the normal functionality of T and dendritic cells as well as the anti-tumor response^1^. In this context, it could be interesting to evaluate whether the increment of anti-tumor response mediated by NC410 can depend also on restoring of the NK cell function.

## IRp60/CD300a

IRp60/CD300a is an inhibitory receptor belonging to CD300 family, a set of genes clustered on chromosome 17 coding for receptors, predominantly expressed on leukocytes, able to generate inhibitory and activating signals regulating different immune processes, such as phagocytosis, cytokine release, proliferation and diseases (80, 106–112). In addition to NK cells, IRp60 is broadly expressed in cells of myeloid or lymphoid origin such as neutrophils (113), eosinophils (106), mast cells (114), pDC (113), B and T cells (115). IRp60 is expressed by the majority of blood NK cells but at higher level in CD56^*bright*^ subset (50). Curiously it has been observed an age-dependent increase of IRp60 expression on NK cells that, in CMV seropositive donors, is associated with increase of CD56^*dim*^ NK cells co-expressing CD57 (116).

IRp60 is a type I transmembrane protein with a single extracellular Ig V-like domain and a long cytoplasmic tail with three canonical ITIMs whose phosphorylation is required for the transmission of the inhibitory signal (50, 117). This inhibitory signal is able to strongly reduce NK cell cytotoxicity induced via different non-HLA-specific or HLA-specific activating receptors (50). IRp60 recognizes phosphatidylserine (PS) and phosphatidylethanolamine (PE), two aminophospholipids exposed on plasma membrane of activated, infected, transformed or apoptotic cells (107, 118–121). Expression of PS on tumor cells has been demonstrated to protect different tumor cell lines from NK cell mediated cytotoxicity (119). Moreover, IRp60 also binds non-lipid molecules such as the human adenovirus-D47 E3/49K protein (122).

To date, a clear role in the control of NK functions in hematological or solid tumors has not been described. However, IRp60 mRNA is highly expressed and associated with poor prognosis in AML (123) and in diffuse large B-cell lymphoma (124), it is hypoxia-inducible in primary human monocytes and macrophages (125) and is up-regulated in tumor-associated macrophages in ovarian carcinoma (126).

## Concluding Remarks

The groundbreaking discoveries of an array of inhibitory receptors controlling the function of individual cells or even of the entire immune response provided tools for unprecedented progress in the therapy of cancer. Thus, KIRs recognizing allotypic determinants on cells offered the means to successfully treat patients with high-risk leukemias by the haplo-HSCT, mostly exploiting alloreactivity of donor-derived NK cells. Perhaps more importantly, the use of checkpoint inhibitors revolutioned the clinical outcome of different lethal-cancers, by reactivating “dormant” effectors potentially capable of destroying tumor cells. Other important receptors controlling cell adhesion/migration, tissue retention or blocking effector cell function at the tumor site, are being investigated in preclinical and clinical settings. It is conceivable that a deeper knowledge of inhibitory receptors useful in the control of excessive immune responses or inflammation, but playing a detrimental role in tumors, will offer important clues for identifying the prevalent mechanism of immunosuppression in a given tumor and to apply specific, evidence-based, approaches for cancer immunotherapy. This is particularly relevant if we consider that some inhibitory receptors are characterized by a broad expression, non-restricted only to NK cells. Thus, immunotherapeutic approaches blocking these inhibitory pathways could act on different types of immune cells, allowing to re-establish a correct cross-talk between the cells of the immune system, an event which is the basis of an optimal antitumor response.

## Author Contributions

All authors have provided intellectual contribution to the work and approved it for publication.

## Conflict of Interest

The authors declare that the research was conducted in the absence of any commercial or financial relationships that could be construed as a potential conflict of interest.

## Footnotes

1 www.nextcure.com/pipeline
